# Intestinal stent implantation using a water injection device with carbon dioxide and transparent cap: A case report

**DOI:** 10.1097/MD.0000000000036330

**Published:** 2023-12-01

**Authors:** Changxiong Wang, Jianye Wu, Xiaoqin Zhang, Xianbao Lu

**Affiliations:** a Department of Digestive, Lishui Hospital of Traditional Chinese Medicine, Lishui, China.

**Keywords:** colonoscopy, colorectal cancer, intestinal obstruction, stents

## Abstract

**Rationale:**

Preoperative endoscopic intestinal stent placement can relieve the symptoms of malignant bowel obstruction (MBO) pending investigations, staging, and surgery, but it is a technically challenging procedure. This paper presents a woman with MBO who successfully underwent intestinal stent implantation using a water injection device with carbon dioxide and a transparent cap.

**Patient concerns:**

We reported a technique for endoscopic intestinal stent placement. A 60-year-old female patient was admitted for abdominal pain and poor bowel movement for 10 days. Computed tomography at a local hospital suggested local stenosis.

**Diagnoses:**

A transparent cap was placed in front of a gastroscope and was used to cross part of the stenotic segment, with water being injected to fill the intestinal cavity continuously. An angiographic catheter was sent along the yellow zebra guidewire passing through the stenotic segment. After exchanging for a colonoscope, a 12-cm intestinal stent was placed along the guidewire.

**Interventions:**

The physician used a single-person water injection-assisted colonoscopy technique in combination with a carbon dioxide gas pump to assist with the air insufflation for colonoscope insertion through the lumen and repeatedly injected water solution to ensure a transparent colonoscopic view.

**Outcomes:**

No intraoperative or postoperative complications were observed. One week after endoscopic intestinal stent placement, the patient underwent radical left hemicolectomy for colon cancer and release of bowel adhesion. The postoperative pathology revealed adenocarcinoma with perineural invasion. The patient recovered well after surgery.

**Lessons:**

Single-person intestinal stent implantation using a water injection device with carbon dioxide and a transparent cap can achieve endoscopic intestinal stent placement for MBO.

## 1. Introduction

Colorectal cancer with comorbid malignant bowel obstruction (MBO) is a common and difficult problem in surgical practice.^[1,2]^ Intestinal stent implantation is a minimally invasive procedure used to treat intestinal strictures or obstructions, and preoperative endoscopic intestinal stent placement can quickly relieve the symptoms of obstruction.^[3,4]^ Still, it is a technically challenging procedure due to the difficulty in finding the luminal orifice and passing a guidewire through.^[5,6]^ In addition, this procedure traditionally requires 2 physicians’ coordination: one using an endoscope for visualization and guidance and another manipulating the wires and placing the stent.^[5–7]^ This paper presents a woman with MBO who successfully underwent intestinal stent implantation using a water injection device with carbon dioxide and a transparent cap. Indeed, this technique allows a single physician to perform the procedure, but intestinal stent placement still needs an assistant. The technique is reported here.

## 2. Case presentation

A 60-year-old female patient was admitted for abdominal pain and poor bowel movement for 10 days. The abdominal enhanced computed tomography at a local hospital suggested local stenosis at the junction of the rectum and sigmoid colon and proximal colon dilatation. After admission, carcinoembryonic antigen was 4.81 ng/mL (reference: 0–5 ng/mL), and carbohydrate antigen 19-9XR was 42.50 U/mL (reference: 0–37 U/mL). Colonoscopy 3 days after admission showed the growth of cauliflower neoplasms into the lumen in the descending colon. Their texture was brittle and easy to bleed, and the lumen was narrow, suggesting a malignant tumor in the descending colon. A biopsy confirmed a moderately differentiated colorectal adenocarcinoma. With the informed consent of the patient, endoscopic intestinal stent placement was performed 5 days after admission.

**Figure 1.**
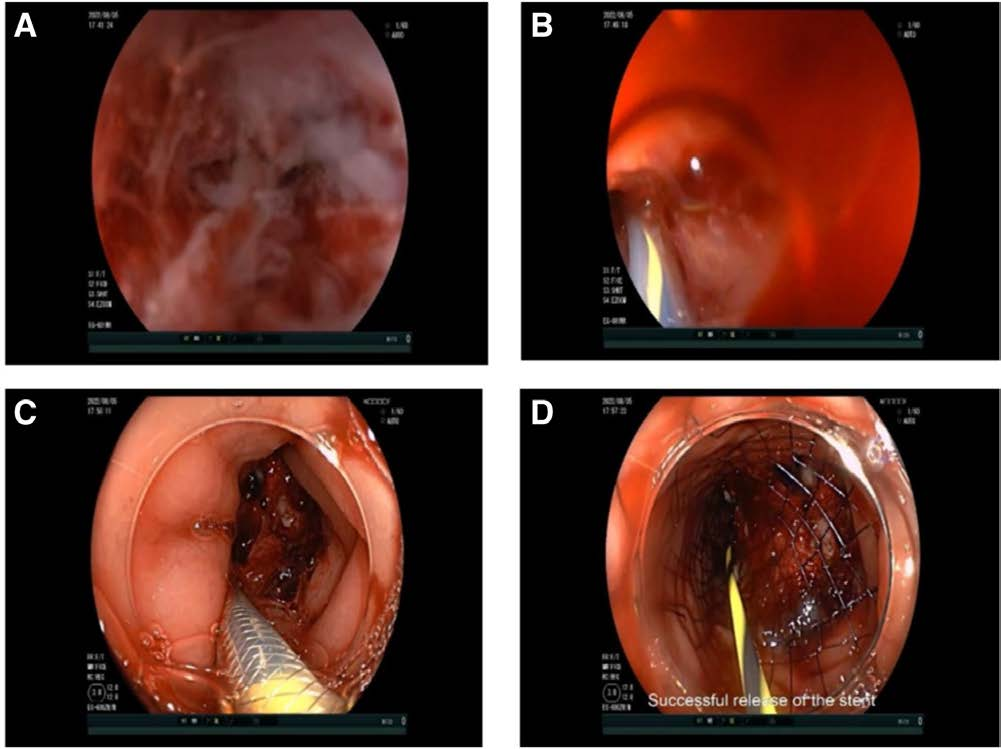
(A) The obvious stenotic fissure underwater. (B) The angiographic catheter follows the guidewire passing through the stenotic segment. (C) The stent was placed along the guidewire. (D) Successful release of the stent.

The patient was kept awake. A transparent cap (XT-DL-128-40; Shangxian Medical Systems Co, Ltd, Shenyang, China) was placed in front of a colonoscope (EC-600ZW/M; FUJINON Corp, Tokyo, Japan). The physician used a single-person water injection colonoscopy technique in combination with a carbon dioxide gas pump (CR4500; AGS Medical Technology Co, Ltd, Hangzhou, China) to assist with the air insufflation for colonoscope insertion through the lumen and repeatedly injected water solution to ensure a clear colonoscopic view. At 3 cm from the anal side of the lower edge of the tumor stenosis, a water solution was injected to fill the intestinal lumen. Due to the long stenotic segment of the tumor, the yellow zebra guide wire (M00556141; Boston Scientific Corp, Natick, MA) failed to pass through. A transparent cap (XT-DL-098-40; Shangxian Medical Systems Co, Ltd) was placed in front of a gastroscope (e.g., -601WR, FUJINON Corp) and was used to cross part of the stenotic segment, with water being injected to fill the intestinal cavity continuously. When the stenotic fissure was clear (Fig. 1A), an angiographic catheter was sent along the yellow zebra guidewire passing through the stenotic segment (Fig. 1B), confirmed by fluoroscopy. Iohexol was injected into the angiographic catheter, and the location and length (approximately 8.5 cm) of the stenotic segment were determined. After exchanging for a colonoscope, a 12-cm intestinal stent (BP-200823; Shiyun Medical Device Corp, Seoul, Korea) was placed along the guidewire (Fig. 1C and D). X-rays showed a good stent position. No intraoperative or postoperative complications, such as gastrointestinal perforation, were observed. One week after endoscopic intestinal stent placement, the patient underwent radical left hemicolectomy for colon cancer and release of bowel adhesion. The surgical procedure was uneventful, and the postoperative pathology revealed a moderately to poorly differentiated adenocarcinoma with perineural invasion. The patient recovered well after surgery and was successfully discharged. The patient was in good condition when writing those lines (about 15 months after surgery).

## 3. Discussion

The case reported here suggests that combining single-person intestinal stent implantation using a water injection device with carbon dioxide and a transparent cap can achieve endoscopic intestinal stent placement.

A previous small case series of 2 patients with MBO, one with Crohn’s disease-related intestinal stenosis and two with gastric outlet malignant stenosis successfully underwent stent placement using a transparent cap and would have been a technical failure if the cap were not used.^[6]^ They indicated that using a transparent cap decreased the time required to cross the stenosis, which is especially important since a prolonged procedure increases the risks of perforation, even when air and/or water are used.^[6]^ In the case reported here, a combination of air insufflation and water injection was first tried to open the stricture but failed. Then, after exchanging for a smaller diameter gastroscope, continuous water injection was successful in opening the lumen, and the stent could be implanted after re-exchanging for a colonoscope. Water injection during colonoscopy has several advantages, including decreased pain and higher safety.^[8,9]^ Since water injection can be used to cure sigmoid volvulus,^[9]^ using it to open an MBO is a logical step. Besides the possible complications of intestinal perforation (e.g., peritonitis), worries about possible cancer cell seeding in case of perforation during MBO opening and stent placement have been raised.^[10]^ The use of a pediatric nasogastroscope (i.e., the gastroscope with the smallest diameter available) has also been reported.^[11,12]^ Therefore, using all possible means to avoid perforation during MBO opening should improve the short- and long-term outcomes of the patients. The intraoperative strategy must be adapted to the patient’s condition and findings.

The transparent cap-assisted technique involves placing a short transparent cap at the tip of an endoscope, which allows for maintaining an appropriate distance between the endoscope lens and the colonic mucosa throughout the examination process.^[9]^ This technique ensures a clear view and enables the cap to push aside the colon folds during endoscope insertion and withdrawal. This technology has low equipment requirements and does not impose an additional burden on patients, making it highly suitable for clinical promotion. For regular colonoscopy, the advantages of transparent cap use include improving the insertion success rate, reducing insertion time, and detecting hidden polyps.^[9]^

The best method to avoid pain during colonoscopy is painless colonoscopy, but it also prevents patient cooperation with the positional changes that can be necessary to pass through the stricture. Moreover, reduced pain perception in patients may increase the risk of complications such as perforation and bleeding because of a lack of feedback from the patient. The discomfort or pain caused by colonoscopy is often due to intestinal distension caused by gas and traction caused by the endoscope. Water exchange colonoscopy effectively avoids excessive dilation, looping, and traction of the colon during insertion by suctioning air and feces from the colonic lumen, thus alleviating pain.^[8,9]^ In addition, repeated flushing during the insertion process cleans the colonic mucosa, facilitating insertion.^[13]^ Water exchange colonoscopy significantly reduces patient pain.^[8,9]^

The strategy proposed here can be performed by a single operator without the need for an assistant, which means 1 physician can perform the transparent cap-assisted water immersion single-person colonoscopy technique, but the intestinal stent placement still needs an assistant. A transparent cap is also inexpensive and safe, especially in the alternative context of a long and eventually unsuccessful procedure that needs to convert to emergent surgery. It can also be easily performed when emerging techniques (e.g., endoscopic ultrasound-guided gastroenterostomy^[14,15]^) are unavailable or contraindicated, or the attending operator lacks experience with them. A two-person approach without fluoroscopy has been described recently,^[7]^ but the single-person method is also feasible, which can be easier to manage and perform in the emergency room. The doctor can guide the endoscope and place the stent alone. The transparent cap provides a clear view, allowing the doctor to observe the location of the lesion and the stent accurately. This method simplifies the procedure and reduces the operation time. Using a stent for managing MBO has disadvantages, including the risk of perforation and the operators’ exposure to radiation.^[7]^ The success rate also depends upon the operator’s experience, the institutional experience, and available resources. Since operator experience is a major factor affecting success.^[7]^ It has been shown that at least 20-30 stent stenting procedures are required to acquire the skills for colonic stent implantation in an emergency setting.^[16,17]^ Nevertheless, stent implantation is an appropriate method with the incidental finding of MBO to buy time to perform the appropriate staging procedures and plan the oncological surgery.

There are few reports on the combined use of a transparent cap and water exchange,^[18]^ and no similar studies have been conducted in China, and not for MBO. The transparent cap-assisted water exchange colonoscopy technique has the following advantages. Alleviating patient pain. In this report, the pain reported by the patient was relatively low, but there was only 1 patient and no control group. Facilitating stricture passage. It has been suggested that this technique facilitates the opening of the colon folds and improves the adenoma detection rate. Therefore, it can also be used to pass through strictures by pushing and opening the colon walls. Reducing the time to pass through the stricture.

In conclusion, this paper proposes that combining single-person intestinal stent implantation using a water injection device with carbon dioxide and a transparent cap can exploit their respective advantages in endoscopic intestinal stent placement and is a safe and beneficial technique.

## Acknowledgments

This work was facilitated by the grant from Key Research and Development Project of Lishui (2022ZDYF23).

## Author contributions

**Data curation:** Changxiong Wang, Xianbao Lu.

**Formal analysis:** Jianye Wu, Xianbao Lu.

**Methodology:** Jianye Wu.

**Writing – original draft:** Changxiong Wang, Xiaoqin Zhang, Xianbao Lu.
